# RepECN: Making ConvNets Better Again for Efficient Image Super-Resolution

**DOI:** 10.3390/s23239575

**Published:** 2023-12-02

**Authors:** Qiangpu Chen, Jinghui Qin, Wushao Wen

**Affiliations:** 1 School of Computer Science and Engineering, Sun Yat-sen University, Guangzhou 510275, China; chenqp8@mail2.sysu.edu.cn (Q.C.); wenwsh@mail.sysu.edu.cn (W.W.); 2 School of Information Engineering, Guangdong University of Technology, Guangzhou 510006, China

**Keywords:** image super-resolution, ConvNet, structural re-parameterization

## Abstract

Traditional Convolutional Neural Network (ConvNet, CNN)-based image super-resolution (SR) methods have lower computation costs, making them more friendly for real-world scenarios. However, they suffer from lower performance. On the contrary, Vision Transformer (ViT)-based SR methods have achieved impressive performance recently, but these methods often suffer from high computation costs and model storage overhead, making them hard to meet the requirements in practical application scenarios. In practical scenarios, an SR model should reconstruct an image with high quality and fast inference. To handle this issue, we propose a novel CNN-based Efficient Residual ConvNet enhanced with structural Re-parameterization (RepECN) for a better trade-off between performance and efficiency. A stage-to-block hierarchical architecture design paradigm inspired by ViT is utilized to keep the state-of-the-art performance, while the efficiency is ensured by abandoning the time-consuming Multi-Head Self-Attention (MHSA) and by re-designing the block-level modules based on CNN. Specifically, RepECN consists of three structural modules: a shallow feature extraction module, a deep feature extraction, and an image reconstruction module. The deep feature extraction module comprises multiple ConvNet Stages (CNS), each containing 6 Re-Parameterization ConvNet Blocks (RepCNB), a head layer, and a residual connection. The RepCNB utilizes larger kernel convolutions rather than MHSA to enhance the capability of learning long-range dependence. In the image reconstruction module, an upsampling module consisting of nearest-neighbor interpolation and pixel attention is deployed to reduce parameters and maintain reconstruction performance, while bicubic interpolation on another branch allows the backbone network to focus on learning high-frequency information. The extensive experimental results on multiple public benchmarks show that our RepECN can achieve 2.5∼5× faster inference than the state-of-the-art ViT-based SR model with better or competitive super-resolving performance, indicating that our RepECN can reconstruct high-quality images with fast inference.

## 1. Introduction

Single Image Super-Resolution (SISR), which aims to reconstruct a high-resolution (HR) image from a low-resolution (LR) image, is an ill-posed problem without one unique solution. As an efficient data-driven technology, deep learning-based SISR methods have shown promising results and achieved better quantitative and qualitative performance than traditional methods. These super-resolution (SR) models can be divided into three categories, including convolutional neural network-based SR methods [1,2], Transformer-based SR methods [3,4], generative adversarial network-based SR methods [5,6].

However, deep learning-based methods require significant computation costs and storage resources to provide high reconstruction accuracy, hindering them from being deployed in resource-limited platforms or scenarios, such as live streaming [7], phone imaging [8], etc. Therefore, an SR model with high super-resolving performance and fast inference is urgently required to meet the requirements of resource-limited scenarios.

**Figure 1:**
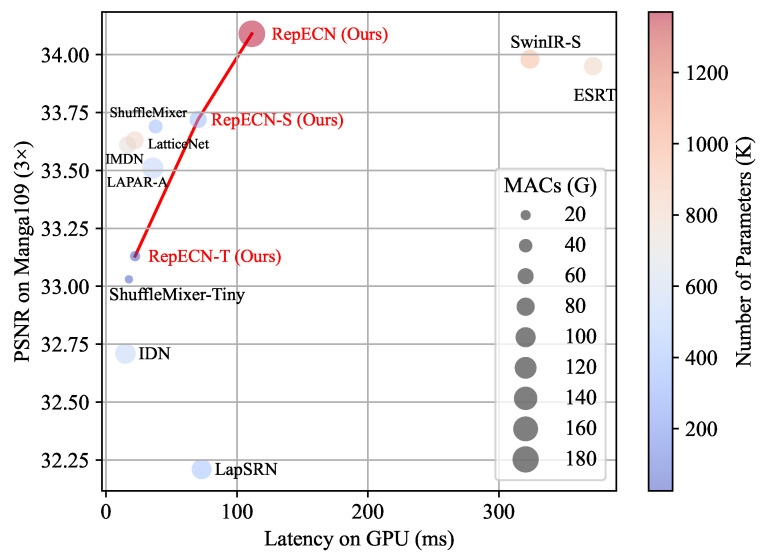
Qualitative trade-off comparison between the performance and the latency of SR models (e.g., SwinIR [4], ESRT [9], ShuffleMixer [13], IDN [19], IMDN [20], LatticeNet [21], LapSRN [22]) on the Manga109 (3×) benchmark dataset. The color normalized mapping represents the model’s parameter number, and the circle’s area represents the Multiply-Accumulates (MACs) of a model. Our proposed models are marked in the red label and line. The comparison results show the superiority of our method.

Lightweight SR models have recently been proposed, but they still face challenges in how to make a better trade-off between inference speed and reconstruction performance. Transformer-based methods, such as SwinIR [4], ESRT [9], and LBNet [10], have shown better performance than CNN-based lightweight models, like ESRN [11], LBFN [12], and ShuffleMixer [13]. However, the multi-head self-attention and encoder–decoder designs overlook the actual inference latency caused by a large amount of memory access cost (MAC) and the parallelism degree of network structure. Our statistical experiments demonstrate that Transformer-based methods suffer from high latencies even with small parameter sizes, as illustrated in Figure 1. In contrast, CNN-based methods infer much faster than other designs with simple structures but suffer from lower reconstruction performance. Thus, ConvNet is often adopted to build efficient and lightweight models for improving inference speed. SR-LUT [14] and SPLUT [15] can reconstruct images faster at the expense of severe performance degradation. Wu et al. [16] explored a compiler-aware SR neural architecture search (NAS) framework to achieve real-time inference on GPU/DSP platforms for mobile devices. However, this work faces difficulties deploying or directly transferring pre-trained models to different hardware platforms with varying instruction architectures. With these considerations, RepSR [17] aims to improve the performance of VGG-like [18] CNN-based models but still has a low-performance cap.

To make a better trade-off between reconstruction performance and inference latency for practical scenarios, we propose a pure CNN-based Efficient Residual ConvNet with structural Re-parameterization (RepECN). The architecture is investigated by the stage-to-block hierarchical design of the ViT-based model to offer both fast speed and high-quality image reconstruction capabilities. The RepECN has three key structural components: a shallow feature extraction module, a deep feature extraction module, and an image reconstruction module. The deep feature extraction module comprises several ConvNet Stages (CNS), each containing six Re-Parameterization ConvNet Blocks (RepCNB), a head layer, and a residual connection. By employing the Transformer-like stage-to-block design, this module allows for learning channel and spatial information by different convolution structures, enabling faster processing speeds, while maintaining similar parameter numbers and performance compared to the Transformer-based models. In addition, we propose a novel image reconstruction module based on nearest-neighbor interpolation and pixel attention to save parameters and maintain reconstruction performance. The extensive experimental results show that our RepECN can achieve 2.5∼5× faster inference than the state-of-the-art ViT-based SR model with better or competitive super-resolving performance, indicating that our RepECN can achieve a better trade-off between super-resolution quality and inference latency for resource-limited scenarios.

In summary, the main contributions of this paper are as follows:

- We propose an efficient and high-accuracy SR model RepECN to offer fast speed and high-quality image reconstruction capabilities using the Transformer-like stage-to-block design paradigm.
- To further improve performance, we employ a large kernel Conv module inspired by ConvNeXt and an Asymmetric Re-Parameterization technique, which is proven to perform better than other symmetric square Re-Parameterization techniques.
- To save parameters and maintain reconstruction performance, we propose a novel image reconstruction module based on nearest-neighbor interpolation and pixel attention.
- Extensive experimental results show that our RepECN can achieve 2.5∼5× faster inference than the state-of-the-art ViT-based SR model with better or competitive super-resolving performance.

## 2. Related Work

### 2.1. CNN-Based Efficient SR

FSRCNN [2] uses upsampling at the end of the model and optimizes the width and depth of convolutional layers from the pioneering model SRCNN [1]. However, the performance is not competitive nowadays. Inspired by residual learning, VDSR [23] and EDSR [24] were proposed to allow deeper networks and avoid gradient disappearance and degradation problems. Later, a series of SR methods proposed by increasing the depth and width of the network (e.g., RCAN [25], RDN [26]) achieved state-of-the-art (SOTA) performance. However, huge Multiply-Accumulates (MACs) and parameters limit their deployment on hardware-limited platforms. To solve this problem, some SR methods [19,20,21,27] focus on improving efficiency. IDN [19] and IMDN [20] use a channel-splitting strategy to reduce computational complexity with redundant parameters. Luo et al. [21] utilize the proposed lattice block to combine residual blocks and introduce a network LatticeNet for fast and accurate SR. MIPN [27] polymerizes multi-scale image features extracted by convolutions with different kernel sizes. The MAI 2021 Challenge [28] brings some extremely lightweight model works [29,30] with real-time inference latency. However, most are optimized for specific NPU mobile platforms, while the SR performance is insufficient. Wu et al. [16] use a neural architecture search (NAS) framework with adaptive SR blocks to find an appropriate model to achieve real-time SR inference. However, it needs to retrain the model when the environment changes, which cannot be used on new devices directly. Unlike these methods that mainly focus on efficiency, we aim at the trade-off of latency and accuracy.

### 2.2. Transformer-Based Efficient SR

Dosovitskiy et al. [31] firstly applied a vision transformer to image recognition. Since then, high-accuracy image SR methods based on transformers became popular. IPT [3] uses a pre-trained vanilla Vision transformer (ViT) on the ImageNet dataset. SwinIR [4] brings Swin Transformer [32] to image restoration tasks and achieves state-of-the-art performance. However, having fewer parameters and MACs does not necessarily result in faster inference latency because other factors, such as memory access cost and degree of parallelism, can also affect latency. The Transformer-based methods suffer from time-consuming and memory-intensive operations, including quadratic-complexity Multi-Head Self-Attention (MHSA) and inefficient non-parallelized window partition. Therefore, some works focus on designing lightweight Transformer-based methods [10,33,34]. A2N [33] obtains lightweight by studying the effectiveness of the attention mechanism. LBNet [10] uses a hybrid network of CNN and Transformer to build an efficient model. SMN [34] simplifies MHSA by separating spatial modulation from channel aggregation, hence making the long-range interaction lightweight. However, there is still potential for improvement in terms of accuracy.

### 2.3. Large Kernel ConvNet

After the introduction of VGG [18], large kernel ConvNets lost popularity due to the higher number of parameters and MACs they require, which is not appropriate for lightweight model designs. However, large kernel convolutions have regained their importance with the development of novel efficient techniques and structures such as transformers and MLPs. Then, ConvMixer [35], ConvNeXt [36], and RepLKNet [37] utilize the large kernel depth-wise convolutions to redesign ConvNet, which achieve competitive performance compared to Transformers. In addition, LKASR [38] also explores the possibility of using a large kernel for lightweight models in the image SR task. However, there is still potential for improvement in terms of SR performance. In this paper, we explore the combination of large kernel convolution and the Structural Re-parameterization technique to further improve performance without a computational cost at the inference phase.

### 2.4. Structural Re-Parameterization

Structural Re-parameterization [39,40,41] equivalently converts model structures via transforming the parameters between training and inference time. These structures enhance the off-the-shelf models without modification of the CNN architecture. Specifically, Ding et al. [39] improve the performance without any inference-time costs by using Asymmetric Convolutional Block (ACB). ACB uses 1D asymmetric convolutions to strengthen the square convolution kernels within a single convolution block. It also uses batch normalizations (BN) [42] in training time to reduce overfitting and accelerate the training process on high-level vision tasks. Besides, Ding et al. [40] designs a more complex version (DBB) that utilizes the symmetric square kernel in the branch during training. DBB performs better in high-level tasks but worse in SR tasks than ACB. RepSR [17] and RMBN [43] use the variants of DBB on VGG-like CNN for SR. However, the SR quality of RepSR is much lower than Transformer-based models. RepSR also introduces the artifacts problem when using BN in a VGG-like SR model. This paper explores the usage of asymmetric structural re-parameterization with BN on large kernel convolutions for image SR.

## 3. Methods

In this section, we first outline the architecture of the proposed Efficient Residual ConvNet with structural Re-parameterization (RepECN) and then introduce the ConvNet Stages (CNS), Re-Parameterization ConvNet Blocks (RepCNB), and the lightweight upsampling module.

### 3.1. Network Architecture

**Figure 2:**
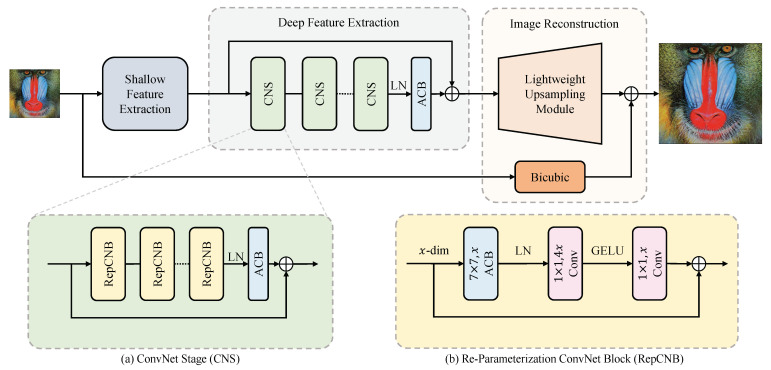
The architecture of the Efficient Residual ConvNet with structural Re-parameterization (RepECN).

We leverage the high-performance, Transformer-like stage-to-block design paradigm and lower computation cost of a pure convolution structure to explore the efficient and high-accuracy network for image super-resolution. As shown in Figure 2, RepECN mainly consists of three modules: shallow feature extraction, deep feature extraction, and high-quality image reconstruction. Different demands of the network sizes employ the same structure, while only different in the number of CNS and backbone channels. The network should also be doing well on other tasks of image resolution.

#### 3.1.1. Shallow and Deep Feature Extraction

Given a low-resolution (LR) image input $I_{LR}\in R^{H\times W\times C_{in}}$ (*H*, *W*, and $C_{in}$ are the numbers of the LR image height, width, and input channels, respectively), we use $A_{SF}\left(\cdot \right)$ to denote an ACB with a 3×3 kernel size. The corresponding shallow feature $O_{0}\in R^{H\times W\times C}$ is extracted as

(1) $$O_{0}=A_{SF}\left(I_{LR}\right),$$

where *C* is the number of output feature channels. Such ACB enhances the standard square-kernel convolution layer. So, it provides a better and simple way to map the input low-dimensional image space to a high-dimensional feature space than conventional shallow feature extraction. In the next module, we extract the deep feature $O_{DF}\in R^{H\times W\times C}$ from $O_{0}$ as

(2) $$O_{DF}=F_{DF}\left(O_{0}\right),$$

where $F_{DF}\left(\cdot \right)$ denotes the entire deep feature extraction module, which consists of *K* ConvNet Stages (CNS), a LayerNorm (LN), and an ACB. Specific for Equation (2), the intermediate outputs $\left\{O_{1},O_{2},\ldots ,O_{K}\right\}$ of CNS and the final output $O_{F}$ of the entire feature extraction module are calculated stage-by-stage as

(3) $$\begin{array}{cc} O_{i} & =F_{CNS_{i}}\left(O_{i-1}\right),\;\;i=1,2,\ldots ,K,\\ O_{DF} & =A_{DF}\left(LN\left(O_{K}\right)\right)\\ O_{F} & =O_{DF}+O_{0},\;\; \end{array}$$

where $F_{CNS_{i}}$ is the *i*-th CNS and $A_{DF}$ is an ACB with a 3×3 kernel at the end of the module. Such an ACB could bring the inductive bias into the depth-wise ConvNet-based network, which helps aggregate shallow and deep features. Meanwhile, the long skip connection aggregates the shallow and deep features, bringing the low-frequency information directly to the next module.

#### 3.1.2. Image Reconstruction

The input LR image has the most primitive information, which should guide the reconstruction output. Additionally, bicubic interpolation can upsample the LR image directly and maintain the original information. Considering that, we reconstruct the super-resolution (SR) image $I_{SR}$ as

(4) $$I_{SR}=U_{F}\left(O_{F}\right)+U_{LR}\left(I_{LR}\right),$$

where $U_{F}\left(\cdot \right)$ and $U_{LR}\left(\cdot \right)$ denote the upsampling of the extracted feature and the bicubic interpolation of the LR image, respectively. The benefit of the aggregation is that the backbone network could focus on learning the high-frequency information of tuning the conventional upsampling of the LR image to a high-qualitative SR image. The upsampling of the extracted feature is implemented by nearest-neighbor interpolation, ACBs, and pixel attention (PA) described in Section 3.3.

#### 3.1.3. Loss Function

The parameters of our network are optimized by $smooth_{L_{1}}$ loss

(5) $$L=\left\{\begin{array}{cc} 0.5\times {∥I_{SR}-I_{HR}∥}_{2},\;\; & if\;{∥I_{SR}-I_{HR}∥}_{1}<1\\ {∥I_{SR}-I_{HR}∥}_{1}-0.5,\;\; & otherwise \end{array}\right.$$

where $I_{HR}$ denotes the corresponding ground-truth HR image, and $I_{SR}$ is the output of RepECN that takes $I_{LR}$ as the input. The $smooth_{L_{1}}$ loss converges faster than the naive $L_{1}$ pixel loss.

### 3.2. ConvNet Stages

The ConvNet Stages (CNS) is a residual block consisting of six Re-Parameterization ConvNet Blocks (RepCNBs), a LayerNorm, and an ACB, as shown in Figure 2a. Each CNS of Equation (3) takes a feature as the input. For the specific *i*-th CNS, we use $O_{i,0}$, taking the place of input $O_{i-1}$ for convenience. Inside such CNS, we obtain intermediate outputs $\left\{O_{i,1},O_{i,2},\ldots ,O_{i,L}\right\}$ by *L* RepCNBs as

(6) $$O_{i,L}=F_{RepCNB_{i,j}}\left(O_{i,j-1}\right),\;\;j=1,2,\ldots ,L,$$

where $F_{RepCNB_{i,j}}\left(\cdot \right)$ denotes the *j*-th RepCNB. Then, a RepCNB is added before the residual connect. The total output of *i*-th CNS is formulated as

(7) $$O_{i}=F_{ACB_{i}}\left(LN\left(O_{i,L}\right)\right)+O_{i,0},$$

where $F_{ACB_{i}}\left(\cdot \right)$ is the ACB at the end of the *i*-th CNS. The ACB could be treated as a standard convolution, while the RepCNB consists of depth-wise and point-wise convolutions. The standard convolution with a small and spatially invariant filter brings a different vision, which benefits the translational equivariance. In addition, the residual connection aggregates different hierarchies of features to let the block fit more complex feature mappings.

#### 3.2.1. Re-Parameterization ConvNet Blocks

The Re-Parameterization ConvNet Blocks (RepCNB) are based on a residual block inspired by the ConvNeXt [36]. The main difference is that we use ACB to enhance the square convolution kernel inside RepCNB. As shown in Figure 2b, given an input with *x* channels, a RepCNB first uses a depth-wise ACB with a 7×7 kernel to extract a feature with the *x* channels. A layer normalization (LN) layer is added behind it. Then, two point-wise convolutional layers are added to learn features across the channel before the residual connection, with GELU non-linearity between them. The first point-wise layer accepts the output of LN with an *x* channel as the input and obtains a feature with 4x channels. The corresponding second point-wise layer takes the feature above as input and obtains the final output with *x* channels.

#### 3.2.2. Asymmetric Convolutional Block

**Figure 3:**
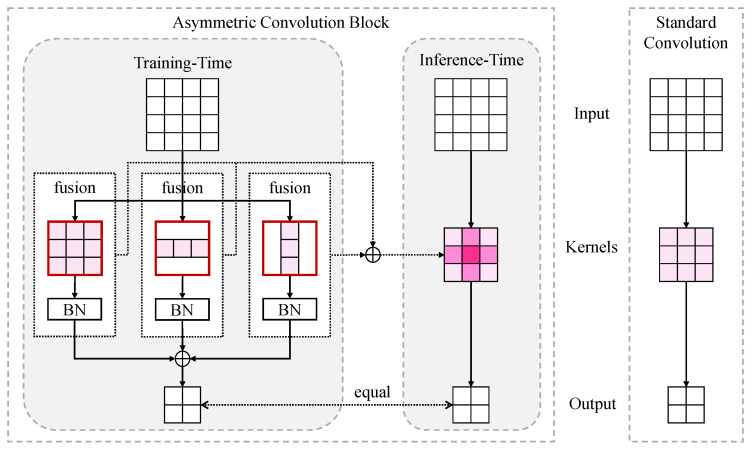
The comparison between Asymmetric Convolutional Block (ACB) and standard Convolution.

An asymmetric Convolutional Block (ACB) is a block using the structural re-para-meterization technique [39], the same as a standard convolution at inference time while different at training time. Figure 3 compares standard convolution (Conv) and ACB with a kernel size of 3×3. The ACB or Conv takes a feature $I_{ACB}$ as the input. At training time, ACB uses three no-bias convolutional layers $\left\{F_{conv_{1}},F_{conv_{2}},F_{conv_{3}}\right\}$ with kernel sizes of 3×3, 1×3, and 3×1, respectively. After batch normalization (BN) for each convolutional layer above, ACB obtains the output $O_{ACB}$ by merging three outputs by element-wise summation as

(8) $$O_{ACB}=\sum_{c=1}^{3}\left(\left(F_{conv_{c}}\left(I_{ACB}\right)-\mu_{c}\right)\frac{\gamma_{c}}{\sigma_{c}}+\beta_{c}\right)$$

where $\mu_{c}$, $\sigma_{c}$, $\gamma_{c}$, and $\beta_{c}$ denote the channel-wise mean, standard deviation, learned scaling factor, and bias term, respectively, while $\sum_{c=1}^{3}$ means element-wise summation for several features. At inference time, ACB first merges channel-wise BN with Conv kernel by BN fusion and then merges three Conv by branch fusion as

(9) $$\begin{array}{cc} O_{ACB} & =\sum_{c=1}^{3}\left(I_{ACB}\times \left(\frac{\gamma_{c}}{\sigma_{c}}K_{c}\right)-\frac{\mu_{c}\gamma_{c}}{\sigma_{c}}+\beta_{c}\right)\\ \; & =I_{ACB}\times \sum_{c=1}^{3}\frac{\gamma_{c}}{\sigma_{c}}K_{c}-\sum_{c=1}^{3}\left(\frac{\mu_{c}\gamma_{c}}{\sigma_{c}}+\beta_{c}\right),\\ K_{inf} & =\sum_{c=1}^{3}\frac{\gamma_{c}}{\sigma_{c}}K_{c},\;\;b_{inf}=\sum_{c=1}^{3}\left(\frac{\mu_{c}\gamma_{c}}{\sigma_{c}}+\beta_{c}\right) \end{array}$$

where $K_{c}$ denotes the kernel of no-bias convolutional layer $F_{conv_{c}}$. The ACB is finally converted to a standard convolutional layer with kernel $K_{inf}$ and bias $b_{inf}$.

### 3.3. Lightweight Upsampling Module

**Figure 4:**
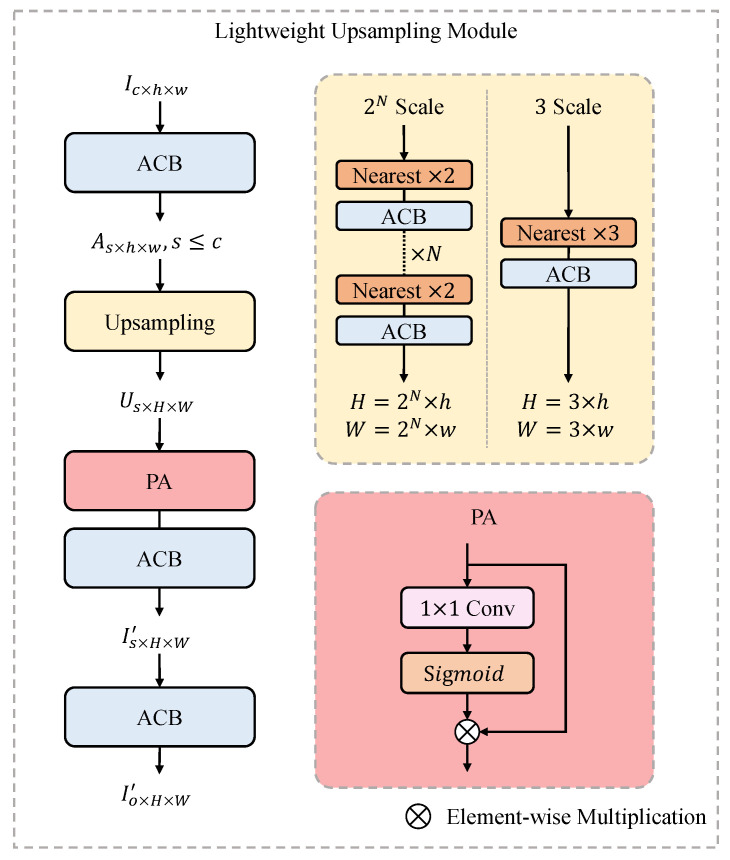
Illustration of the proposed upsampling module.

As shown in Figure 4, we choose the nearest-neighbor interpolation to upsample the input feature, followed by an ACB. Rather than sub-pixel convolution like pixel shuffle, such upsampling choice saves the parameter number without performance degradation. We first use an upsampling operation to transfer the feature $O_{F}$ from the entire feature extraction module in Equation (3). The upsampling operation consists of several pairs of nearest-neighbor interpolation and ACB. Each pair only upsamples on scale factor 2 or 3, limiting the whole module to accept scale factor $2^{N}$ or 3. The module should support varying scale factors by adopting the interpolation scale factor. Then, inspired by PAN [44], we employ a pixel attention (PA) layer and an ACB to reconstruct the SR feature. The PA can enhance the reconstruction and improve the SR quality. Finally, a second ACB layer generates the output $U_{F}\left(O_{F}\right)$ of the upsampling module in Equation (4).

## 4. Experiments

This section uses several commonly used benchmark datasets to compare the proposed network with effective and state-of-the-art SISR models. In addition, some ablation studies are used to analyze the rationality of our proposed modules.

### 4.1. Experimental Settings

#### 4.1.1. Datasets and Indicators

We train the proposed network using the DIV2K dataset [45] while validating it on the Set5 [46] dataset. The 800 training and 100 validation image pairs in DIV2K are used as the training dataset. The indicators of evaluation for SISR performance are peak signal-to-noise ratio (PSNR) [47] and structural similarity index (SSIM) [48] on benchmark datasets Set5, Set14 [49], B100 [50], Urban100 [51], and Manga109 [52]. We use MATLAB to calculate them on the Y channel of the YCbCr space converted from the RGB space of the image.

#### 4.1.2. Training Details

We group the efficient models into three level sizes according to the parameter number. The parameter number of extremely tiny, small, and base size is smaller than 100 K, 500 K, and 1500 K, respectively. The settings of the training hyperparameters for our RepECN-T (tiny), RepECN-S (small), and RepECN (base) models are described in Table 1. The RepCNB and channel in the table denote the RepCNB number in each CNS and the channel number of each intermediate feature, while the patch denotes the size of RGB patches cropped from LR images as the input. The total training epochs of RepECN-T, RepECN-S, and RepECN are set to 3000, 2000, and 1500, respectively. Each minibatch comprises 32 patches for training all three models. The learning rate is set to $2\times 10^{-4}$ and reduced by half at $\left[\frac{1}{2},\frac{4}{5},\frac{9}{10},\frac{19}{20}\right]$ of the total epoch.

The latency of inference on the CPU and GPU platform are measured for generating a 720P SR image (the width and height are 1280×720) on an Intel Xeon Gold 5118 CPU (12 cores, 2.30 GHz, and 6 load-data threads) and Nvidia Titan V (12 GB of HBM2 memory and 5120 CUDA cores) GPU acceleration, respectively. Each latency takes an average of 50 running results. The multiply-accumulates (MACs) are also measured for generating a 720P SR image (1280×720).

### 4.2. Experimental Results

#### Performance and Latency Comparison

To show the effectiveness of our RepECN fairly, we chose the state-of-the-art Transformer-based models with similar parameter numbers, which are trained on the same DIV2K dataset. Table 2 shows the quantitative performance comparisons between the proposed RepECN and state-of-the-art Transformer-based models: SwinIR [4], ESRT [9], and LBNet [10]. As for the models with parameter numbers less than 1500 K, RepECN achieves the best or second-best performance on five benchmark datasets for three standard scale factors with much less latency. Specifically, compared to the state-of-the-art SwinIR-S with similar PSNR/SSIM, RepECN only needs one-fifth of the latency for a scale factor 2 on the platform with GPU. Especially, LBNet and ESRT cannot do inference for a scale factor of 2 on our platform with GPU because of memory resource limitations.

To show the high SR quality of our RepECN structure, we chose the current CNN-based models in different sizes of parameter numbers. Specially, the training dataset of ShuffleMixer and LAPAR is DF2K (a merged dataset with DIV2K [45] and Flickr2K [53]), which contains much more image pairs. Table 3 shows the quantitative performance comparisons between the proposed RepECN and CNN-based models: SRCNN [1], FSRCNN [2], ShuffleMixer [13], IDN [19], IMDN [20], LatticeNet [21], LapSRN [22], EDSR [24], DRRN [54], and LAPAR [55]. Our RepECN family achieves state-of-the-art performance in all tiny, small, and base sizes. Specifically, RepECN-T (less than 100 K) outperforms ShuffleMixer-Tiny with a 0.45 dB gain on Urban100 (2×). RepECN-S (less than 500 K) outperforms ShuffleMixer with a 0.41 dB gain on Urban100 (2×) using similar parameter numbers. In addition, RepECN-S also outperforms LatticeNet with a 0.06 dB gain on Urban100 (2×) using about half the parameter numbers. It proves that our design of ConvNet outperforms all of the previous designs. In conclusion, our model achieves state-of-the-art performance with a better trade-off between inference speed and performance.

**Figure 5:**
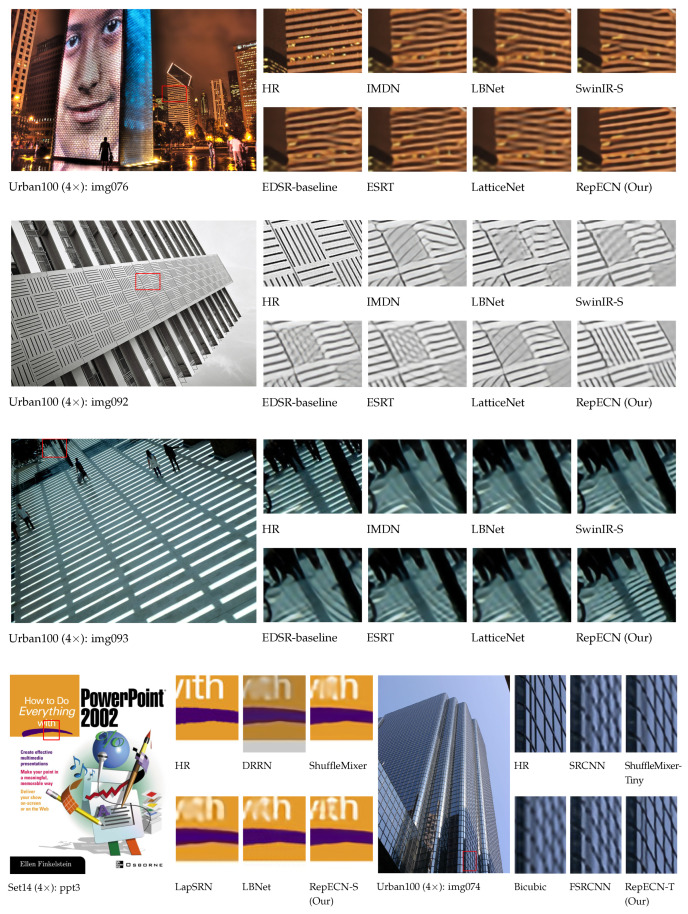
Visual qualitative comparison of the efficient state-of-the-art models (e.g., SwinIR-S [4], ESRT [9], LBNet [10], IMDN [20], LatticeNet [21], EDSR-baseline [24]) on Set14 [49] and Urban100 [51] benchmark datasets for 4× single image super-resolution (SISR). Zoom in for the best view.

**Figure 6:**
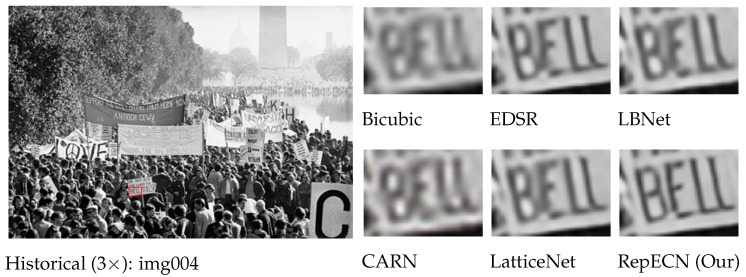
Visual qualitative comparisons on a real-world historical image dataset for 3× SR. The proposed ALAN generates a cleaner view than other methods (e.g., LBNet [10], LatticeNet [21], EDSR [24], CARN [56]) with fewer artifacts.

To evaluate our RepECN qualitatively, we also show visual comparisons in Figure 5, including three different sizes of RepECN and the corresponding size state-of-the-art models for scale factor 4 SISR on benchmark images. All three sizes of RepECN can restore higher frequency detailed textures and alleviate the blurring artifacts with more visually pleasing images. In contrast, most other models produce incorrect textures with blurry artifacts. Furthermore, we evaluate our model on real LR images from a historical dataset [22], as shown in Figure 6. RepECN can generate smoother details with a clearer structure than other models. This indicates the high effectiveness of our proposed RepECN.

### 4.3. Ablation Study and Analysis

For the ablation study, we train RepECN family models on DIV2K [45] with 1000 epochs for 2× SISR in Section 4.3.1, Section 4.3.3 and Section 4.3.4, progressively adding useful elements to construct RepECN-T. Then, we train RepECN with CNSs, RepCNBs, channels, and epochs setting to 4, 6, 60, and 1500 epochs for 2× SISR as the baseline model and modify the first three hyperparameters individually in Section 4.3.5. In addition, we train FSRCNN variants on DIV2K [45] with 3000 epochs for 2× SISR in Section 4.3.2. In all sections, the performance comparison uses the PSNR on benchmark dataset Set5 [46].

#### 4.3.1. Impact of Normalization in CNS and ACB

To explore the effect of layer normalization (LN) in each CNS, we remove the head layer in CNS and batch normalization (BN) inside ACB to reduce their effects. Table 4 firstly shows that LN is necessary for better performance, as the SR quality of RepECN-T-A is lower than RepECN-T-B and RepECN-T-C. Then, the table illustrates that LayerNorm before the residual connection in CNS further improves the PSNR than LN after the residual connection.

In addition, we compare using batch normalization (BN) inside ACB. The no-BN variant RepECN-T-C skips normalization and generates a bias for each convolutional layer in ACB during training. When switching to inference, add the weight and bias of three convolutional layers in ACB to the single convolutional layer used as the inference ACB layer. Table 4 shows that the normalization inside ACB is important as RepECN-T-D improves the PSNR performance by 0.01 dB. Apart from that, the training with normalization in ACB will not converge when removing the residual connection of LR input to the output while using the pixel shuffle upsampling.

#### 4.3.2. Impact of Structural Re-Parameterization

To demonstrate the effectiveness of structural re-parameterization for image super-resolution (SR), we trained multiple variants of FSRCNN, a model with ample room for improvement. We first replace the upsampling module of FSRCNN with our proposed lightweight upsampling module as a variant FSRCNN-N, which improves the PSNR performance by 0.31. Then, we use a symmetric square kernel structural re-parameterization technique DBB [40] for each ConvNet layer in FSRCNN-N, a similar but more complex technique as used in RepSR [17]. FSRCNN-N-DBB can improve the SR performance by a 0.16 dB gain on PSNR. Finally, we replace the DBB with the asymmetric kernel structural re-parameterization technique ACB. FSRCNN-N-ACB further improves the SR quality by a 0.09 dB gain on PSNR. In conclusion, structural re-parameterization can improve the performance of CNN-based SR models, while the asymmetric kernel technique is better than a symmetric square one.

#### 4.3.3. Impact of the Head Layer in CNS

The effect of using a head layer (the last ACB before the residual connection) in CNS is shown in Table 4. The base version RepECN-T is designed as one 3×3 ACB. With this version, the performance gains on PSNR by 0.4 dB. Furthermore, the table shows that one 3×3 ACB is better than three 3×3 ACB (whose channel number of the second layer is one-fourth of the input and output channel number). RepECN-T-E saves a few parameters (5K) with 0.02 dB performance degradation on PSNR compared to RepECN-T. To achieve higher performance, we finally choose to use one 3×3 ACB as the head layer in CNS.

#### 4.3.4. Impact of Nearest-Neighbor Interpolation with Pixel Attention in Upsampling Module

Table 4 and Table 5 show the performance improvement of the proposed upsampling module in Section 3.3 with pixel attention (PA). In Table 4, The pixel shuffle of the variant RepECN-T-G is the same as the image reconstruction module in SwinIR [4]. The nearest-neighbor without PA of variant RepECN-T-F removes the PA block from the proposed upsampling module. The table shows that the nearest-neighbor interpolation saves parameters with performance improvement, and the PA is necessary, as it improves the PSNR by 0.02 dB. Table 5 shows that the proposed upsampling module can significantly improve the performance of FSRCNN by a 0.31 dB gain on PSNR.

#### 4.3.5. Impact of CNS, RepCNB, and Channel Numbers

**Figure 7:**
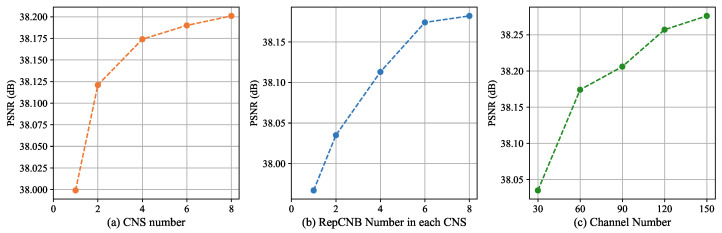
Ablation study on different number settings of the RepECN structure. The illustrations are tested on Set5 [46] for 2× SISR.

The effects of CNS numbers, RepCNB numbers in each CNS, and channel numbers of each layer are shown in Figure 7, respectively. We observed that the performance is positively correlated with such three hyper-parameters. In addition, as the number of settings increases, the performance growth tends to flatten out. As a result, it is a trade-off between the performance and the model’s size. To achieve high performance and fast inference, we choose the point with the maximum change in slope as the setting. Especially, the RepCNB number of each CNS is fixed to 6, as the performance is more sensitive when reducing it than the others.

## 5. Conclusions and Future Works

In this paper, we propose a pure CNN-based SR model, Efficient Residual ConvNet with structural Re-parameterization (RepECN), with fast speed and high quality. The model contains three modules: shallow feature extraction, deep feature extraction, and image reconstruction. We borrow the stage-to-block hierarchical design of the ViT-based model to keep the SOTA performance using ConvNet. Specifically, we proposed the ConvNet Stages (CNS) for deep feature extraction. Each CNS comprises six Parameterization ConvNet Blocks (RepCNB), a basic ACB, a LayerNorm, and a residual connection. We also introduce a lightweight upsampling module containing nearest-neighbor interpolation and pixel attention, which saves parameters without performance degradation. We evaluate the proposed RepECN for different sizes on commonly used benchmark datasets. Experiments show that RepECN achieves state-of-the-art performance while providing much faster inference than Transformer-based models and much better performance than CNN-based models.

## Figures and Tables

**Table 1:** Hyperparameter settings of different-sized RepECN.

Model	CNS	RepCNB	Channel	Patch	Epoch
RepECN-T	2	6	24	64×64	3000
RepECN-S	3	6	42	64×64	2000
RepECN	5	6	60	48×48	1500

**Table 2:** The performances of PSNR (dB) and SSIMs on standard benchmark datasets for our RepECN models trained on DIV2K compared with Vision Transformer-based models. The best and second-best SR performances are marked in red and blue, respectively. Blanked entries denote unavailable.

				#Latency		Set5		Set14		BSD100		Urban100		Manga109	
**Methods**	**Scale**	**#Params**	**#MACs**	**GPU(ms)**	**CPU(s)**	**PSNR**	**SSIM**	**PSNR**	**SSIM**	**PSNR**	**SSIM**	**PSNR**	**SSIM**	**PSNR**	**SSIM**
LBNet-T	×2	407K	22.0 G	-	241.29	37.95	0.9602	33.53	0.9168	32.07	0.8983	31.91	0.9253	38.59	0.9768
ESRT	×2	677K	161.8 G	-	55.00	38.03	0.9600	33.75	0.9184	32.25	0.9001	32.58	0.9318	39.12	0.9774
LBNet	×2	731K	153.2 G	-	314.27	38.05	0.9607	33.65	0.9177	32.16	0.8994	32.30	0.9291	38.88	0.9775
RepECN-S (Ours)	×2	411K	117.5 G	145.2	2.96	38.10	0.9607	33.68	0.9187	32.24	0.9004	32.30	0.9301	38.76	0.9773
SwinIR-S	×2	878K	195.6 G	1074.3	13.61	38.14	0.9611	33.86	0.9206	32.31	0.9012	32.76	0.9340	39.12	0.9783
RepECN (Ours)	×2	1262K	336.5 G	242.6	6.66	38.20	0.9612	33.85	0.9199	32.32	0.9013	32.68	0.9337	39.11	0.9777
LBNet-T	×3	407K	22.0 G	1551.5	49.80	34.33	0.9264	30.25	0.8402	29.05	0.8042	28.06	0.8485	33.48	0.9433
ESRT	×3	770K	82.1 G	372.0	12.62	34.42	0.9268	30.43	0.8433	29.15	0.8063	28.46	0.8574	33.95	0.9455
LBNet	×3	736K	68.4 G	2099.6	65.25	34.47	0.9277	30.38	0.8417	29.13	0.8061	28.42	0.8559	33.82	0.9460
RepECN-S (Ours)	×3	411K	69.9 G	70.3	1.38	34.47	0.9277	30.41	0.8439	29.15	0.8064	28.30	0.8551	33.72	0.9456
SwinIR-S	×3	886K	87.2 G	323.8	5.10	34.62	0.9289	30.54	0.8463	29.20	0.8082	28.66	0.8624	33.98	0.9478
RepECN (Ours)	×3	1262K	185.1 G	111.4	2.82	34.67	0.9291	30.48	0.8459	29.25	0.8089	28.65	0.8628	34.09	0.9482
LBNet-T	×4	410 K	12.6 G	567.5	18.29	32.08	0.8933	28.54	0.7802	27.54	0.7358	26.00	0.7819	30.37	0.9059
ESRT	×4	751K	58.6 G	135.7	4.92	32.19	0.8947	28.69	0.7833	27.69	0.7379	26.39	0.7962	30.75	0.9100
LBNet	×4	742K	38.9 G	714.6	21.83	32.29	0.8960	28.68	0.7832	27.62	0.7382	26.27	0.7906	30.76	0.9111
RepECN-S (Ours)	×4	427K	57 G	45.7	1.03	32.32	0.8964	28.69	0.7833	27.62	0.7375	26.19	0.7889	30.54	0.9099
SwinIR-S	×4	897K	49.6 G	176.1	2.97	32.44	0.8976	28.77	0.7858	27.69	0.7406	26.47	0.7980	30.92	0.9151
RepECN (Ours)	×4	1295K	140 G	72.0	1.98	32.48	0.8985	28.76	0.7856	27.67	0.7395	26.45	0.7971	30.92	0.9139

**Table 3:** The performances of PSNR (dB) and SSIMs on standard benchmark datasets for CNN-based models. The best and second-best SR performances are marked in red and blue, respectively. Blanked entries denote unavailable.

					Set5		Set14		BSD100		Urban100		Manga109	
**Methods**	**Scale**	**Dataset**	**#Params**	**#MACs**	**PSNR**	**SSIM**	**PSNR**	**SSIM**	**PSNR**	**SSIM**	**PSNR**	**SSIM**	**PSNR**	**SSIM**
Bicubic	×2	-	-	-	33.66	0.9299	30.24	0.8688	29.56	0.8431	26.88	0.8403	30.80	0.9339
SRCNN	×2	T91	69K	63.7G	36.66	0.9542	32.45	0.9067	31.36	0.8879	29.50	0.8946	35.60	0.9663
FSRCNN	×2	T91	25K	15.1G	37.00	0.9558	32.63	0.9088	31.53	0.8920	29.88	0.9020	36.67	0.9710
ShuffleMixer-Tiny	×2	DIV2K+Flickr2K	108K	25G	37.85	0.9600	33.33	0.9153	31.99	0.8972	31.22	0.9183	38.25	0.9761
RepECN-T (Ours)	×2	DIV2K	104K	31.6G	37.90	0.9601	33.41	0.9164	32.09	0.8984	31.67	0.9239	38.30	0.9763
LapSRN	×2	DIV2K	435K	146.0G	37.52	0.9591	32.99	0.9124	31.80	0.8952	30.41	0.9103	37.27	0.9740
DRRN	×2	DIV2K	298K	6.8T	37.74	0.9591	33.23	0.9136	32.05	0.8973	31.23	0.9188	37.88	0.9749
IDN	×2	DIV2K	553K	174.1G	37.83	0.9600	33.30	0.9148	32.08	0.8985	31.27	0.9196	38.01	0.9749
EDSR-baseline	×2	DIV2K	1370K	316.2G	37.99	0.9604	33.57	0.9175	32.16	0.8994	31.98	0.9272	38.54	0.9769
IMDN	×2	DIV2K	694K	158.8G	38.00	0.9605	33.63	0.9177	32.19	0.8996	32.17	0.9283	38.88	0.9774
LAPAR-A	×2	DIV2K+Flickr2K	548K	171.0G	38.01	0.9605	33.62	0.9183	32.19	0.8999	32.10	0.9283	38.67	0.9772
ShuffleMixer	×2	DIV2K+Flickr2K	394K	91G	38.01	0.9606	33.63	0.9180	32.17	0.8995	31.89	0.9257	38.83	0.9774
LatticeNet	×2	DIV2K	756K	169.5G	38.06	0.9607	33.70	0.9187	32.19	0.8999	32.24	0.9288	38.93	0.9774
RepECN-S (Ours)	×2	DIV2K	411K	117.5G	38.10	0.9607	33.68	0.9187	32.24	0.9004	32.30	0.9301	38.76	0.9773
RepECN (Ours)	×2	DIV2K	1262K	336.5G	38.20	0.9612	33.85	0.9199	32.32	0.9013	32.68	0.9337	39.11	0.9777
Bicubic	×3	-	-	-	30.39	0.8682	27.55	0.7742	27.21	0.7385	24.46	0.7349	26.95	0.8556
SRCNN	×3	T91	69K	63.7G	32.75	0.9090	29.30	0.8215	28.41	0.7863	26.24	0.7989	30.48	0.9117
FSRCNN	×3	T91	25K	13.6G	33.18	0.9140	29.37	0.8240	28.53	0.7910	26.43	0.8080	31.10	0.9210
ShuffleMixer-Tiny	×3	DIV2K+Flickr2K	114K	12G	34.07	0.9250	30.14	0.8382	28.94	0.8009	27.54	0.8373	33.03	0.9400
RepECN-T (Ours)	×3	DIV2K	104K	19.9G	34.20	0.9259	30.25	0.8405	29.03	0.8031	27.86	0.8453	33.13	0.9419
LapSRN	×3	DIV2K	435K	98.6G	33.81	0.9220	29.79	0.8325	28.82	0.7980	27.07	0.8275	32.21	0.9350
DRRN	×3	DIV2K	298K	6.8T	34.03	0.9244	29.96	0.8349	28.95	0.8004	27.53	0.8378	32.71	0.9379
IDN	×3	DIV2K	553K	105.6G	34.11	0.9253	29.99	0.8354	28.95	0.8013	27.42	0.8359	32.71	0.9381
EDSR-baseline	×3	DIV2K	1555K	160.1G	34.37	0.9270	30.28	0.8417	29.09	0.8052	28.15	0.8527	33.45	0.9439
IMDN	×3	DIV2K	703K	71.5G	34.36	0.9270	30.32	0.8417	29.09	0.8046	28.17	0.8519	33.61	0.9445
LAPAR-A	×3	DIV2K+Flickr2K	544K	114.0G	34.36	0.9267	30.34	0.8421	29.11	0.8054	28.15	0.8523	33.51	0.9441
ShuffleMixer	×3	DIV2K+Flickr2K	415K	43G	34.40	0.9272	30.37	0.8423	29.12	0.8051	28.08	0.8498	33.69	0.9448
LatticeNet	×3	DIV2K	765K	76.3G	34.40	0.9272	30.32	0.8416	29.09	0.8047	28.19	0.8511	33.63	0.9442
RepECN-S (Ours)	×3	DIV2K	411K	69.9G	34.47	0.9277	30.41	0.8439	29.15	0.8064	28.30	0.8551	33.72	0.9456
RepECN (Ours)	×3	DIV2K	1262K	185.1G	34.67	0.9291	30.48	0.8459	29.25	0.8089	28.65	0.8628	34.09	0.9482
Bicubic	×4	-	-	-	28.42	0.8104	26.00	0.7027	25.96	0.6675	23.14	0.6577	24.89	0.7866
SRCNN	×4	T91	69K	63.7G	30.48	0.8628	27.50	0.7513	26.90	0.7101	24.52	0.7221	27.58	0.8555
FSRCNN	×4	T91	25K	13.6G	30.72	0.8660	27.61	0.7550	26.98	0.7150	24.62	0.7280	27.90	0.8610
ShuffleMixer-Tiny	×4	DIV2K+Flickr2K	113K	8G	31.88	0.8912	28.46	0.7779	27.45	0.7313	25.66	0.7690	29.96	0.9006
RepECN-T (Ours)	×4	DIV2K	110K	17.1G	32.05	0.8930	28.52	0.7791	27.52	0.7335	25.84	0.7772	30.09	0.9038
LapSRN	×4	DIV2K	870K	182.4G	31.54	0.8852	28.09	0.7700	27.32	0.7275	25.21	0.7562	29.09	0.8900
DRRN	×4	DIV2K	298K	6.8T	31.68	0.8888	28.21	0.7720	27.38	0.7284	25.44	0.7638	29.45	0.8946
IDN	×4	DIV2K	553K	81.9G	31.82	0.8903	28.25	0.7730	27.41	0.7297	25.41	0.7632	29.41	0.8942
EDSR-baseline	×4	DIV2K	1518K	114.2G	32.09	0.8938	28.58	0.7813	27.57	0.7357	26.04	0.7849	30.35	0.9067
IMDN	×4	DIV2K	715K	40.9G	32.21	0.8948	28.58	0.7811	27.56	0.7353	26.04	0.7838	30.45	0.9075
LAPAR-A	×4	DIV2K+Flickr2K	659K	94.0G	32.15	0.8944	28.61	0.7818	27.61	0.7366	26.14	0.7871	30.42	0.9074
ShuffleMixer	×4	DIV2K+Flickr2K	411K	28G	32.21	0.8953	28.66	0.7827	27.61	0.7366	26.08	0.7835	30.65	0.9093
LatticeNet	×4	DIV2K	777K	43.6G	32.18	0.8943	28.61	0.7812	27.56	0.7353	26.13	0.7843	30.54	0.9075
RepECN-S (Ours)	×4	DIV2K	427K	57G	32.32	0.8964	28.69	0.7833	27.62	0.7375	26.19	0.7889	30.54	0.9099
RepECN (Ours)	×4	DIV2K	1295K	140G	32.48	0.8985	28.76	0.7856	27.67	0.7395	26.45	0.7971	30.92	0.9139

**Table 4:** Ablation study on the several designs of RepECN, including layer normalization in CNS, batch normalization in ACB, head layer in CNS, and upsampling design. The best SR performances are marked in red.

Design Name	LayerNorm	BN in ACB	Head in CNS	Upsampling	Params	PSNR
RepECN-T-A	**✗**	**✗**	**✗**	Nearest (PA)	75K	37.78
RepECN-T-B	After Connect				75K	37.80
RepECN-T-C	Before Connect	**✗**			75K	37.81
RepECN-T-D	Before Connect	**✓**	**✗**		75K	37.82
RepECN-T-E		**✓**	Three 3×3 ACB		99K	37.84
RepECN-T			One 3×3 ACB	Nearest (PA)	104K	37.86
RepECN-T-F			One 3×3 ACB	Nearest (no PA)	103K	37.84
RepECN-T-G				Pixel Shuffle	114K	37.83

**Table 5:** Ablation study on the structural re-parameterization and upsampling design for the simple 3×3 ConvNet model FSRCNN to prove the effectiveness. The best SR performances are marked in red.

Design Name	Upsampling	Re-Parameterization	PSNR
FSRCNN	Deconvolution	**✗**	37.00
FSRCNN-N	Nearest (PA)	**✗**	37.31
FSRCNN-N-DBB	Nearest (PA)	DBB	37.47
FSRCNN-N-ACB	Nearest (PA)	ACB	37.56

## Data Availability

Data is contained within the article. The data presented in this study are available in https://github.com/qpchen/RepECN (accessed on 28 November 2023).
